# Supplementary figures and images for: PERK recruits E-Syt1 at ER–mitochondria contacts for mitochondrial lipid transport and respiration
Source: J Cell Biol. 2023 Feb 23;222(3):e202206008. doi: 10.1083/jcb.202206008 (PMC9998969; doi:10.1083/jcb.202206008)

A

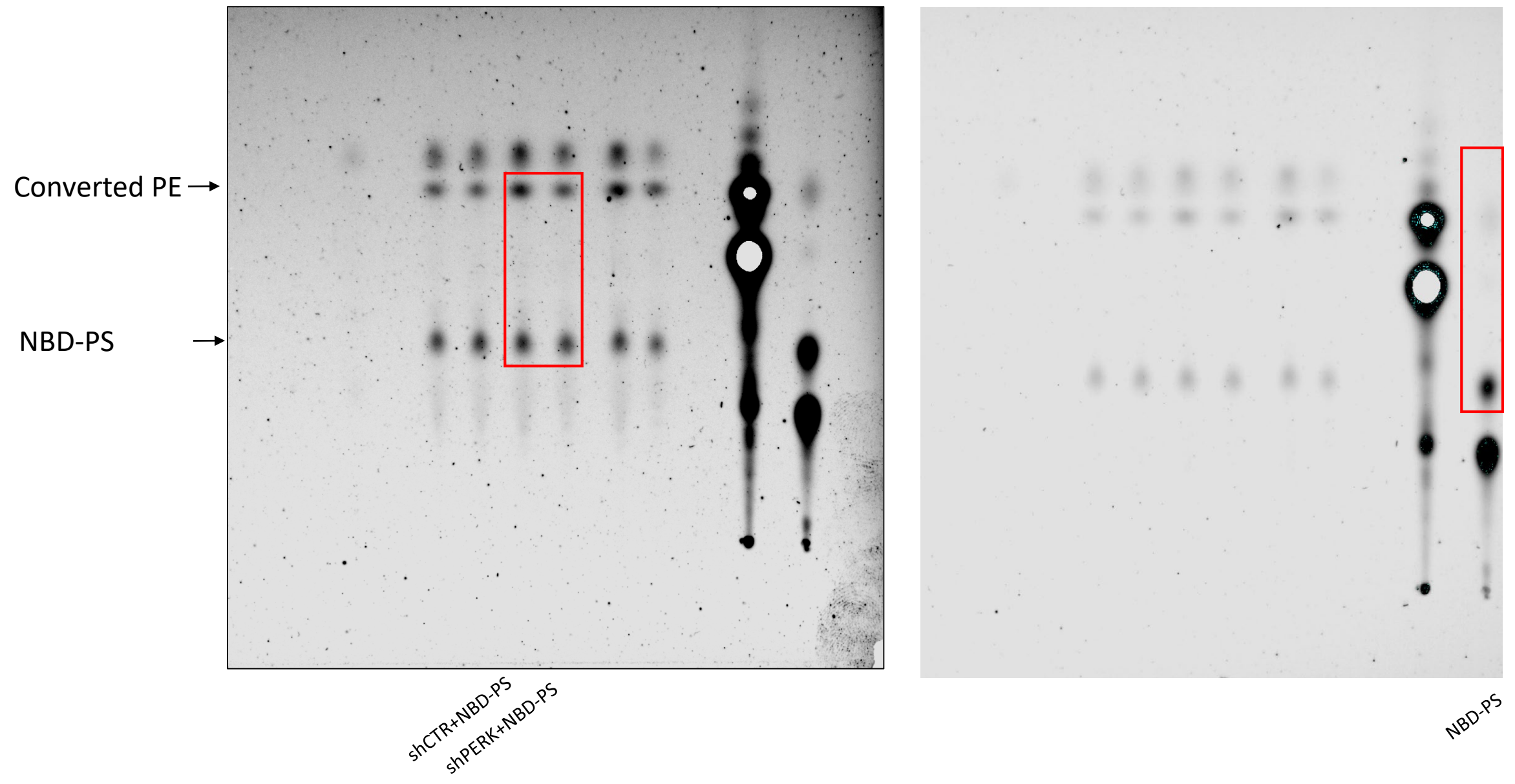

Supplement: SourceData F2 — is the source file for Fig. 2. [file JCB_202206008_SourceDataF2.pdf]

A

Converted PE →

NBD-PS →

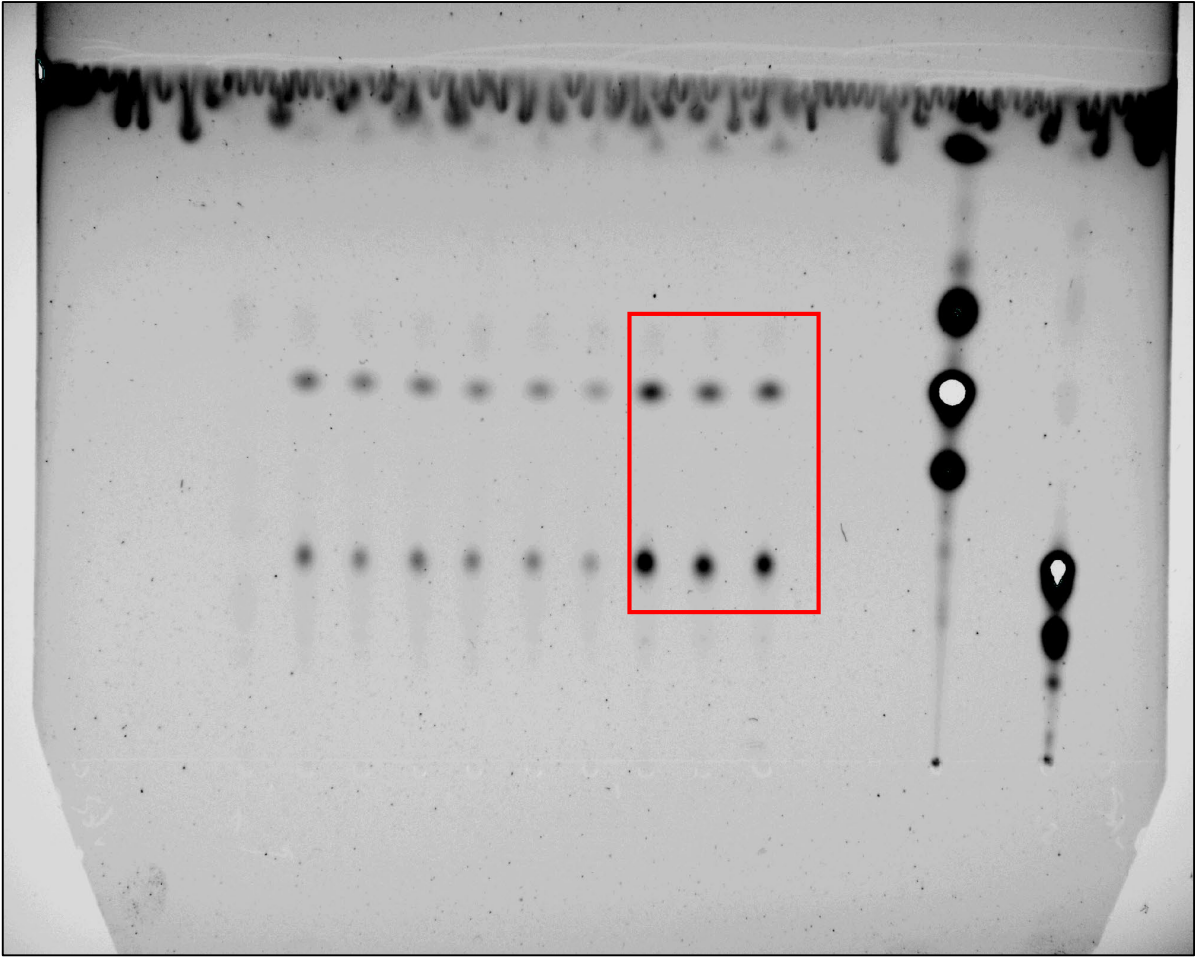

WT+NBD-PS  
DKO+NBD-PS  
DKO+E-Syt1 NBD-PS

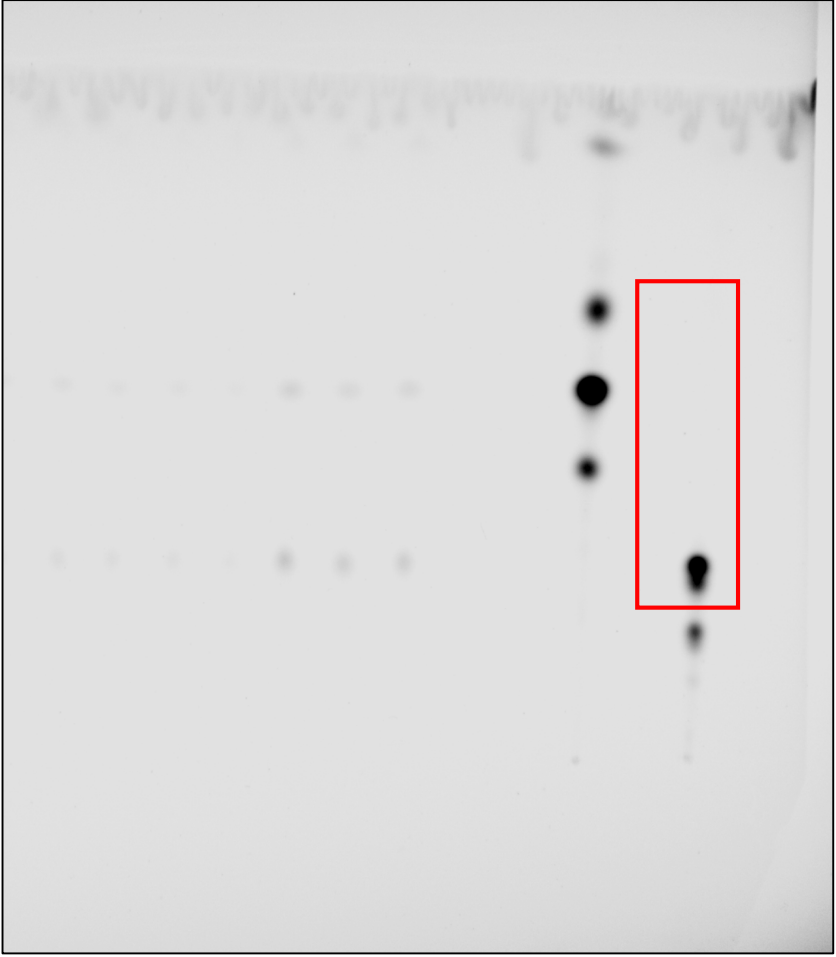

NBD-PS

Supplement: SourceData F5 — is the source file for Fig. 5. [file JCB_202206008_SourceDataF5.pdf]

E

Ab: PERK

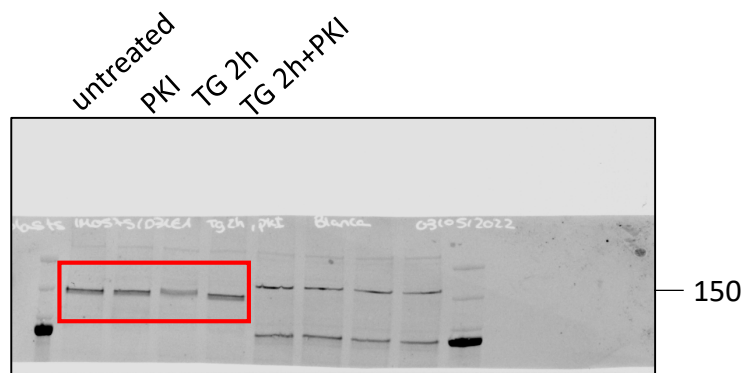

SourceData6

Ab: p-EIF2a

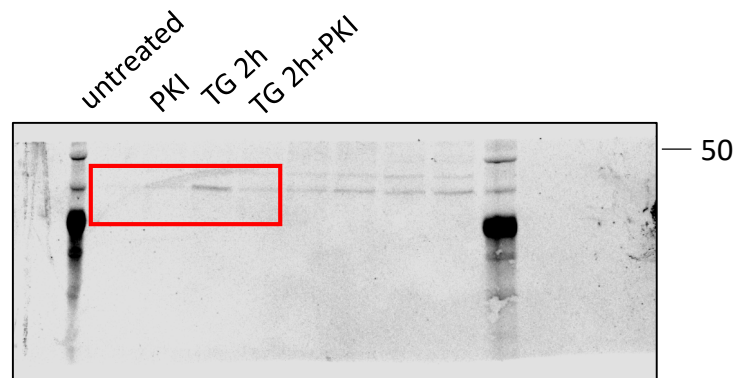

Ab: Tot EIF2a

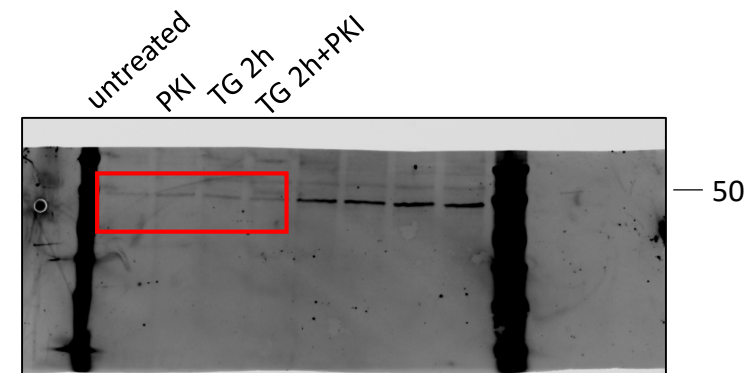

Ab: ACTIN

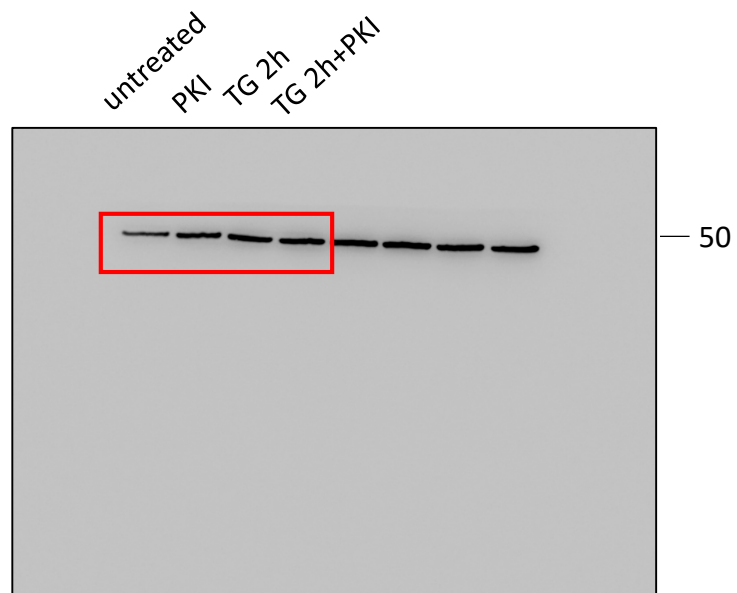

Supplement: SourceData F6 — is the source file for Fig. 6. [file JCB_202206008_SourceDataF6.pdf]

A Ab: IP3R3

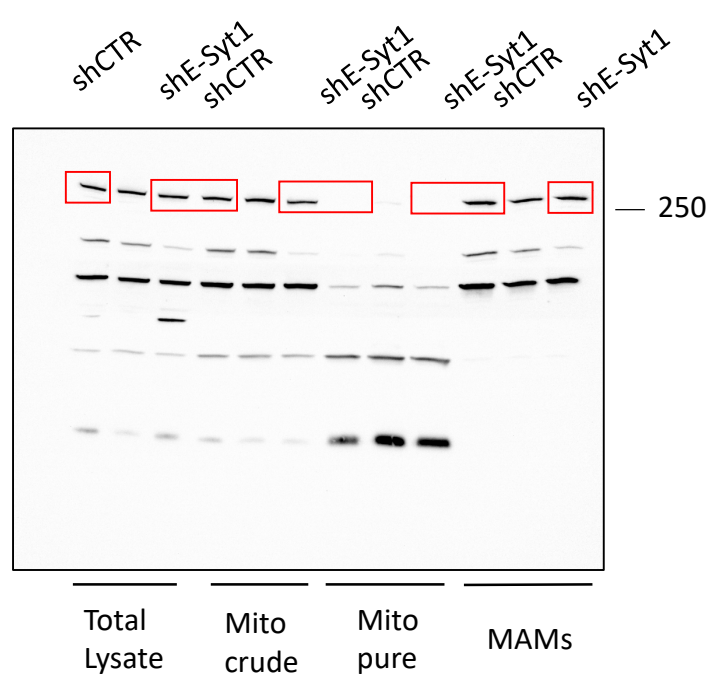

SourceData3S

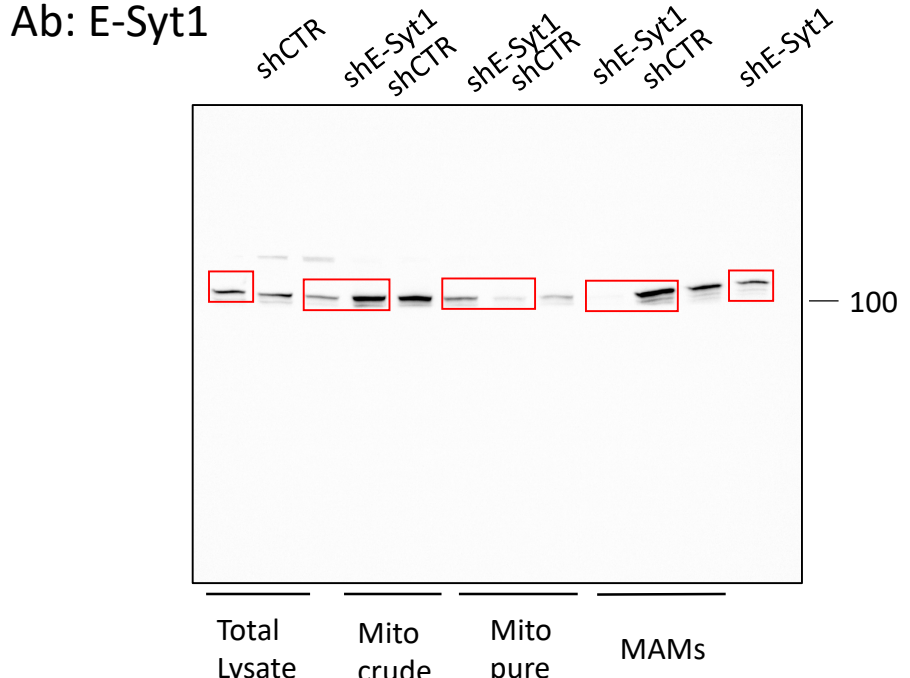

Ab: VDAC1

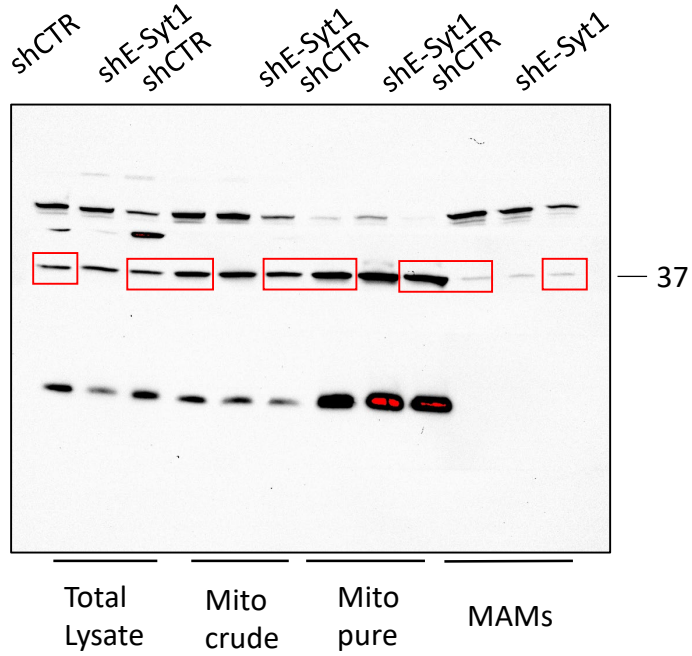

Ab: CNX

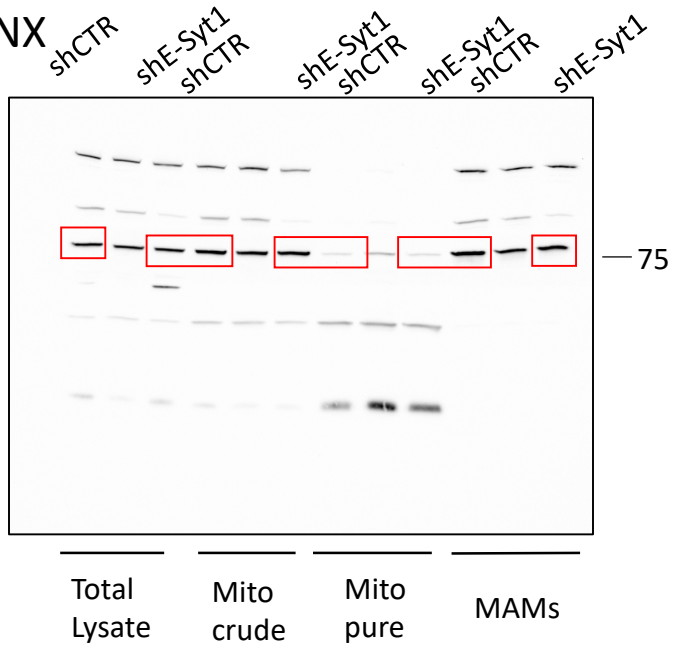

Ab: CYTC

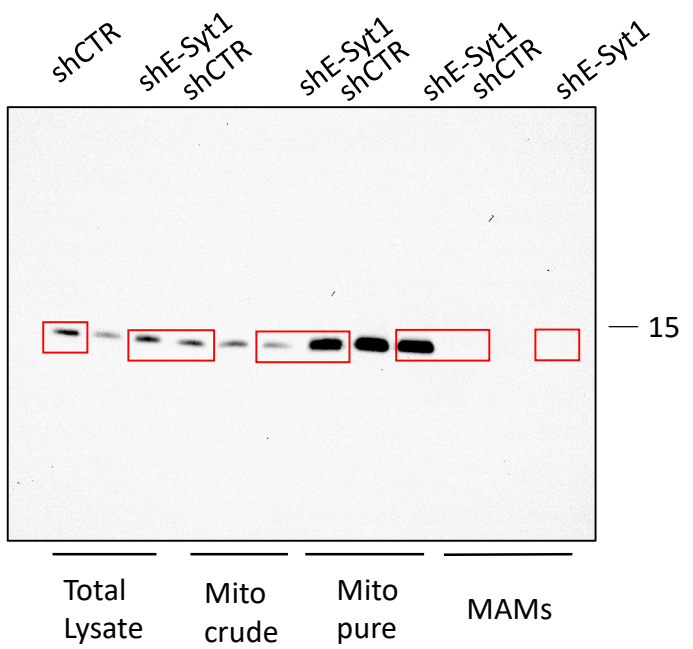

# SourceData3S

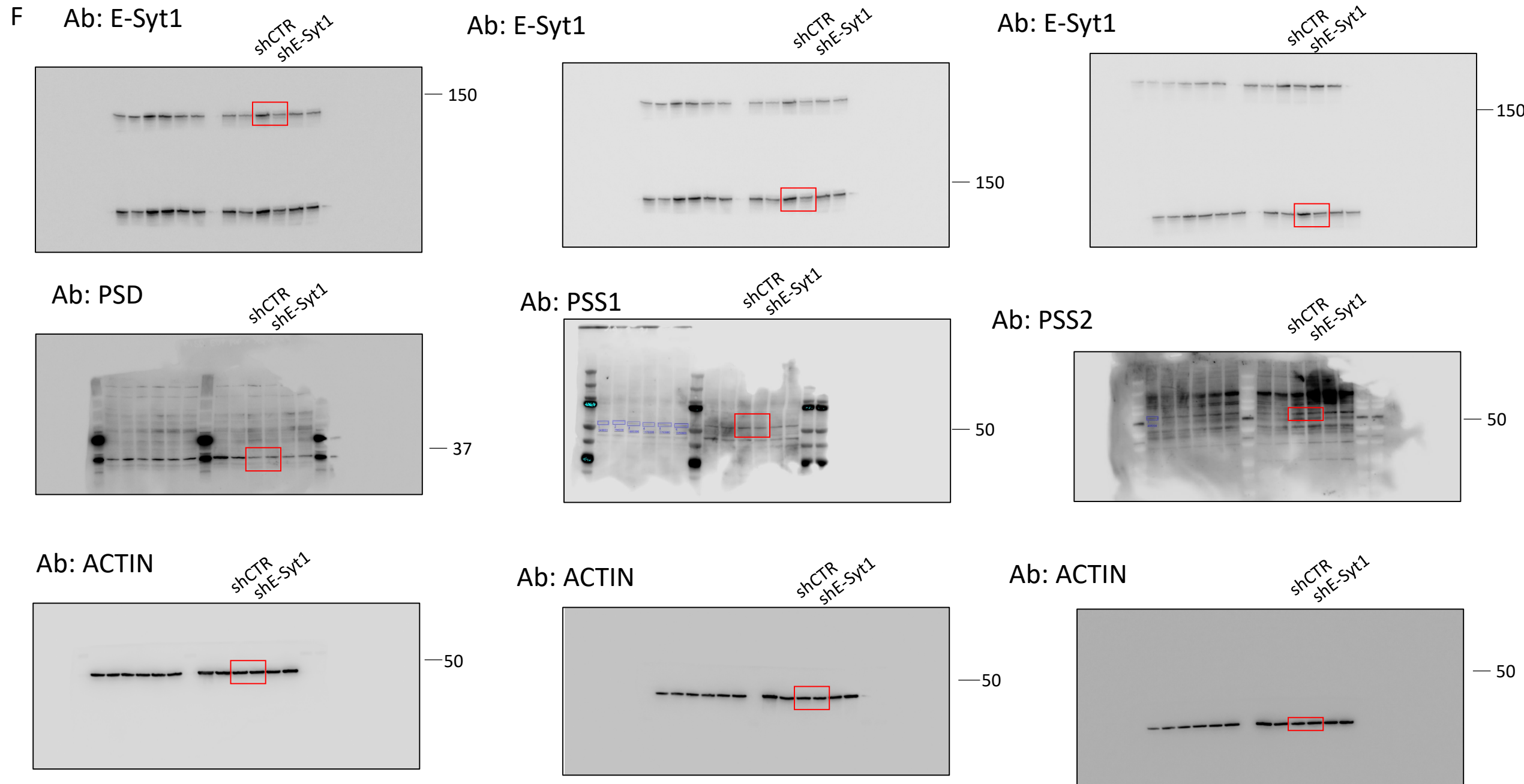

Supplement: SourceData FS3 — is the source file for Fig. S3. [file JCB_202206008_SourceDataFS3.pdf]
