# Supplementary material for: PERK recruits E-Syt1 at ER–mitochondria contacts for mitochondrial lipid transport and respiration
Source: J Cell Biol. 2023 Feb 23;222(3):e202206008. doi: 10.1083/jcb.202206008 (PMC9998969; doi:10.1083/jcb.202206008)

SourceData3

A

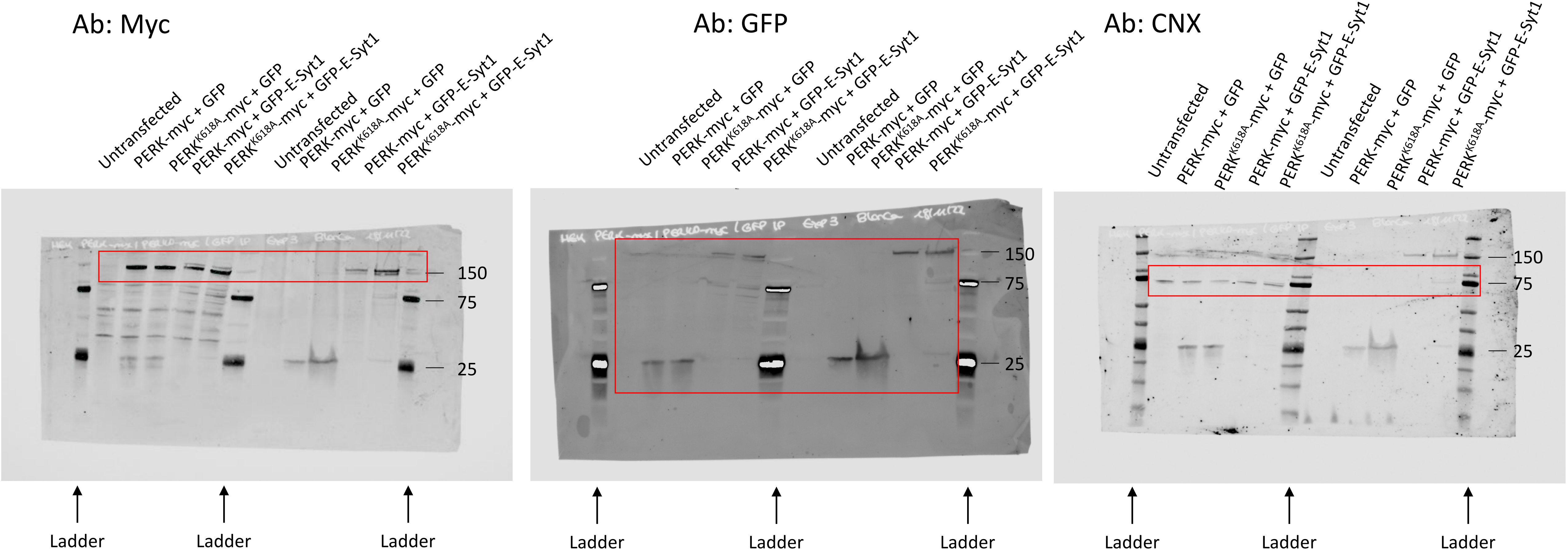

SourceData3

C

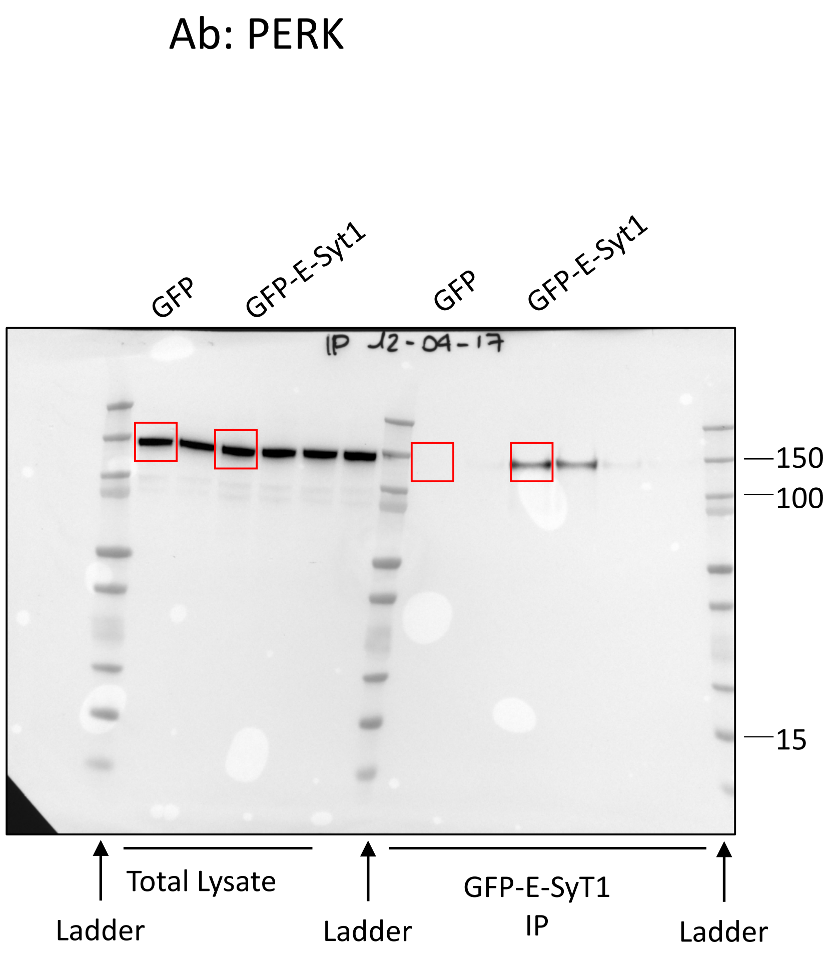

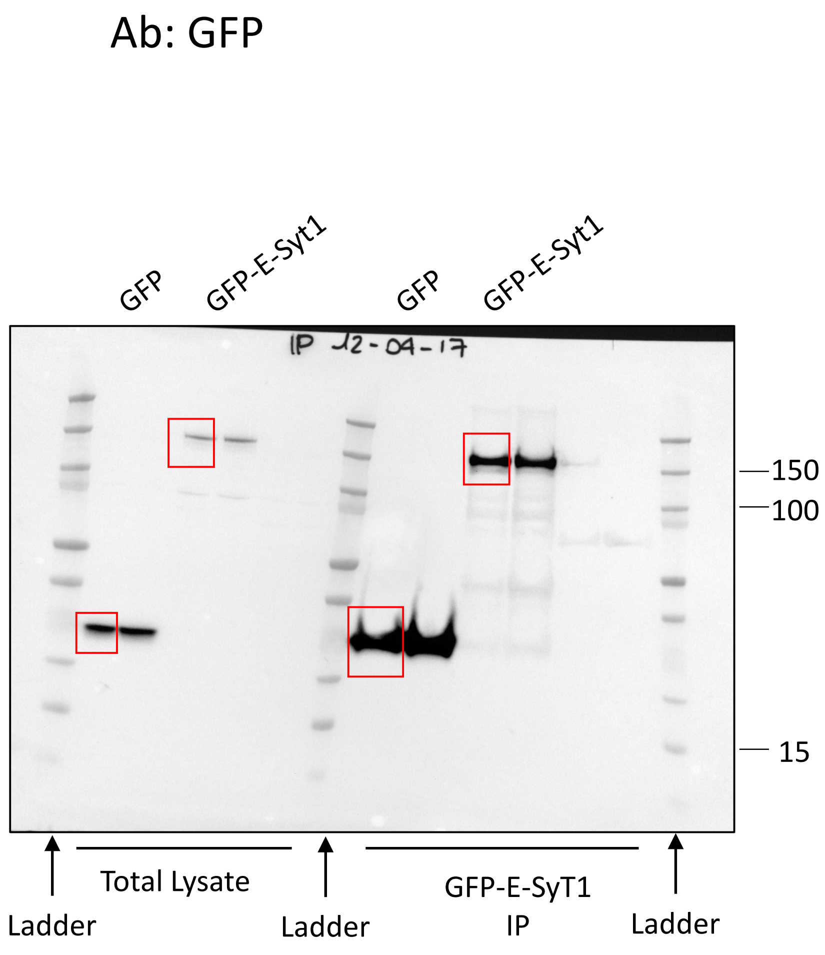

SourceData3

F

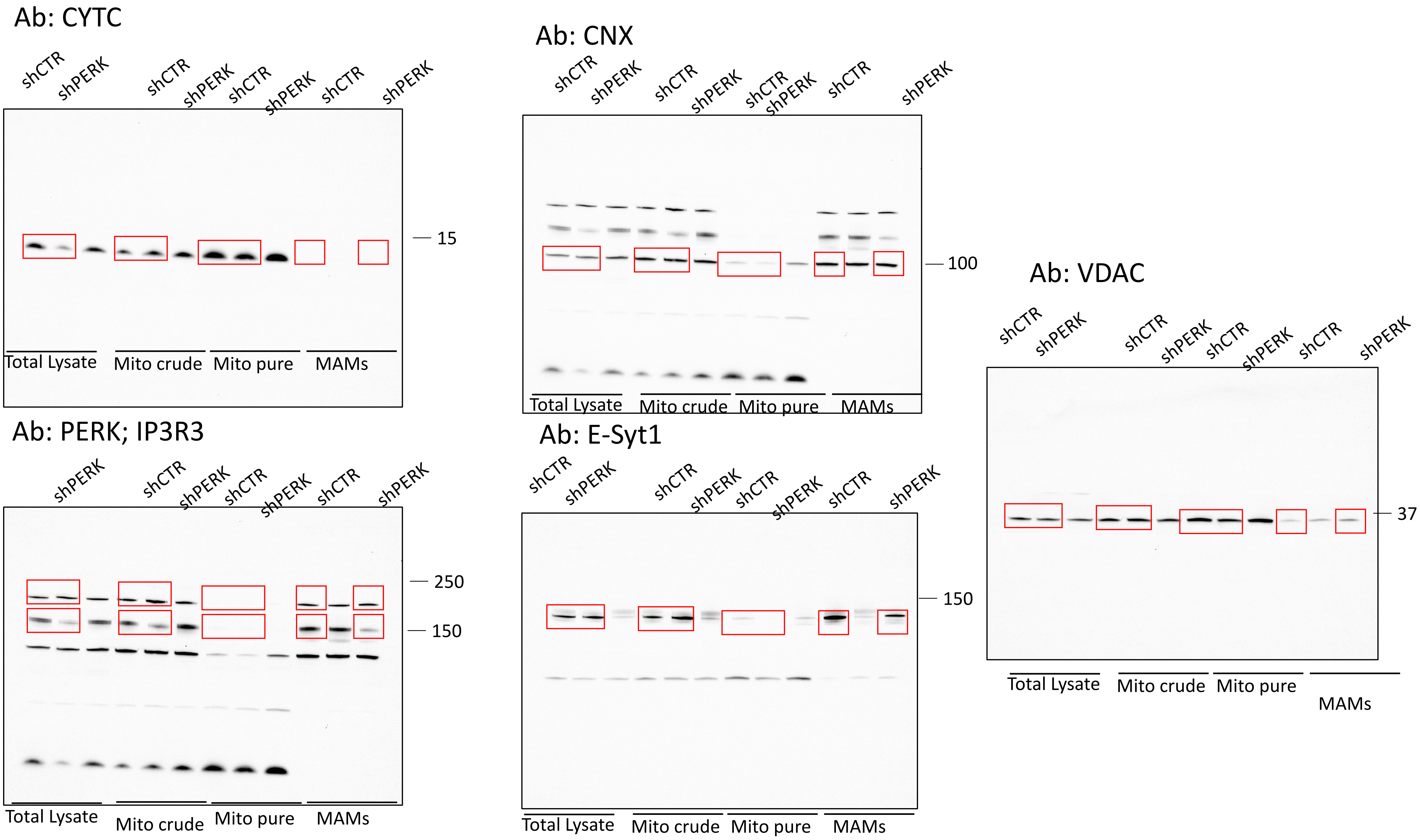

SourceData3

I

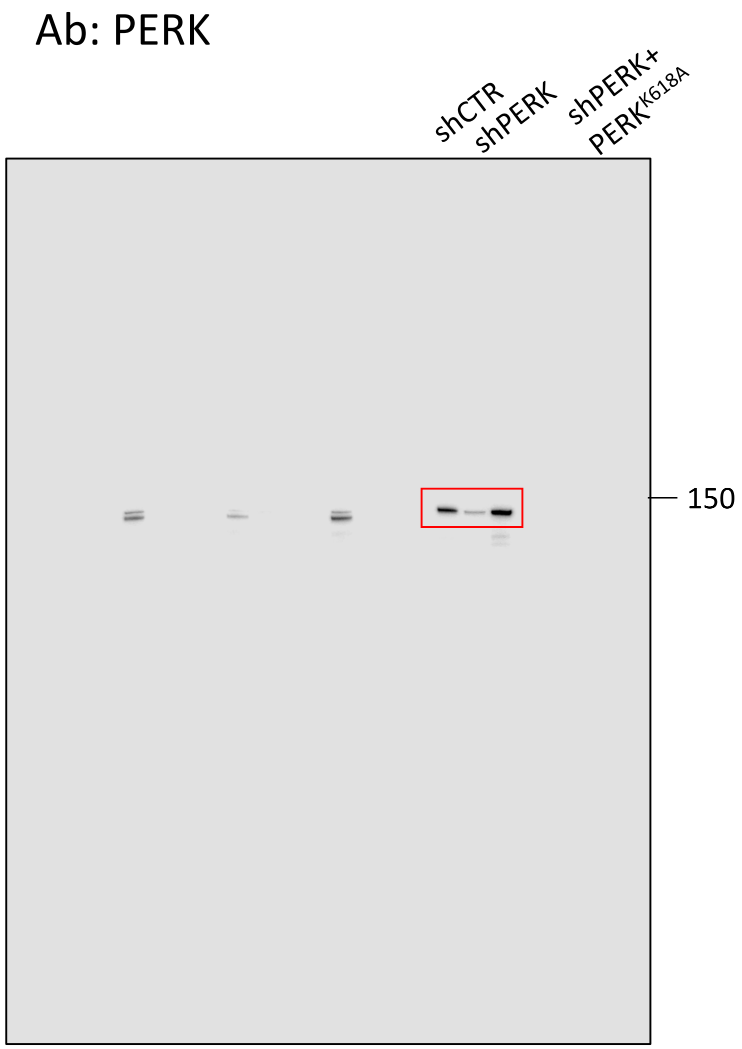

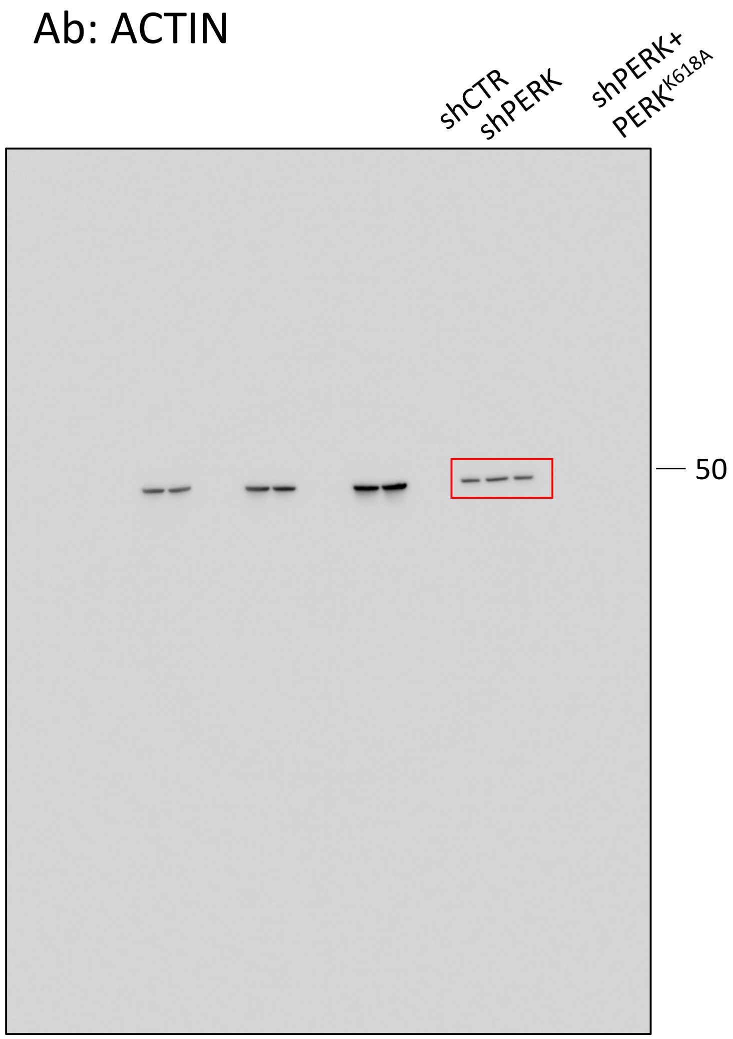

Supplement: SourceData F3 — is the source file for Fig. 3. [file JCB_202206008_SourceDataF3.pdf]
