# Supplementary material for: PERK recruits E-Syt1 at ER–mitochondria contacts for mitochondrial lipid transport and respiration
Source: J Cell Biol. 2023 Feb 23;222(3):e202206008. doi: 10.1083/jcb.202206008 (PMC9998969; doi:10.1083/jcb.202206008)

SourceData4
(MERGED)

A

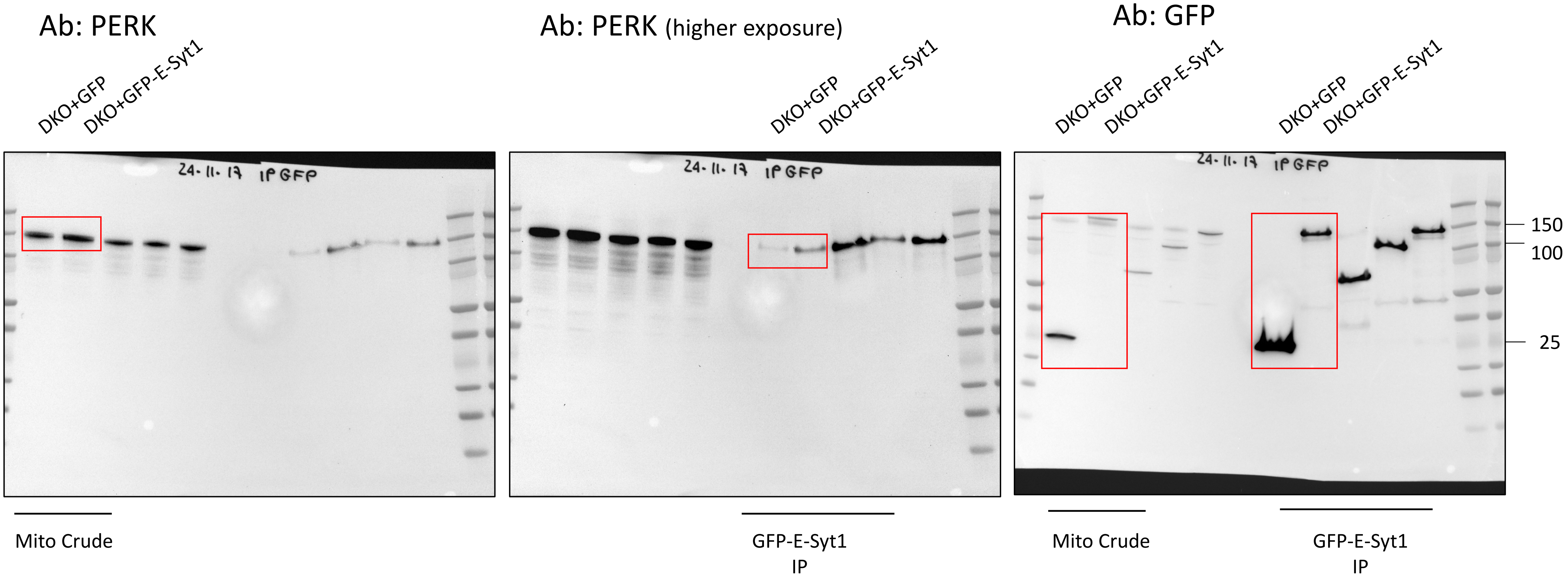

SourceData4
(PERK/GFP NOT merged with Ladder)

A

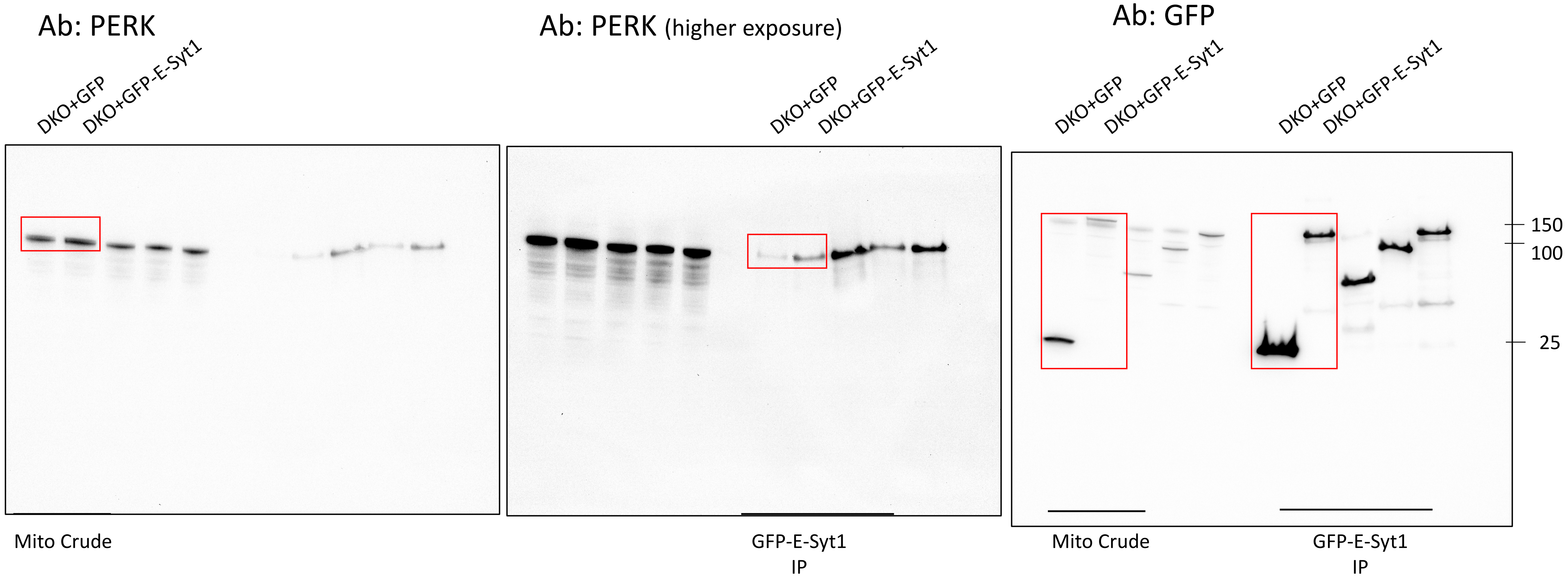

SourceData4
(only Ladder)

A

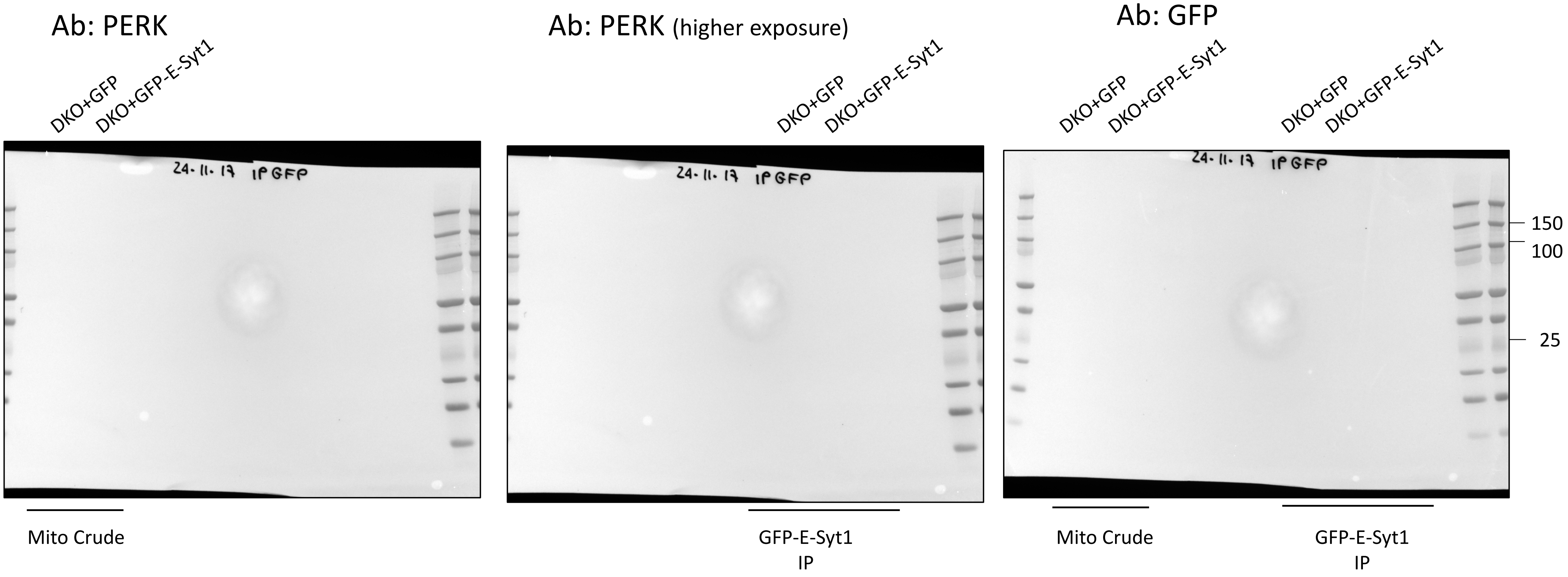

C

SourceData4
(MERGED)

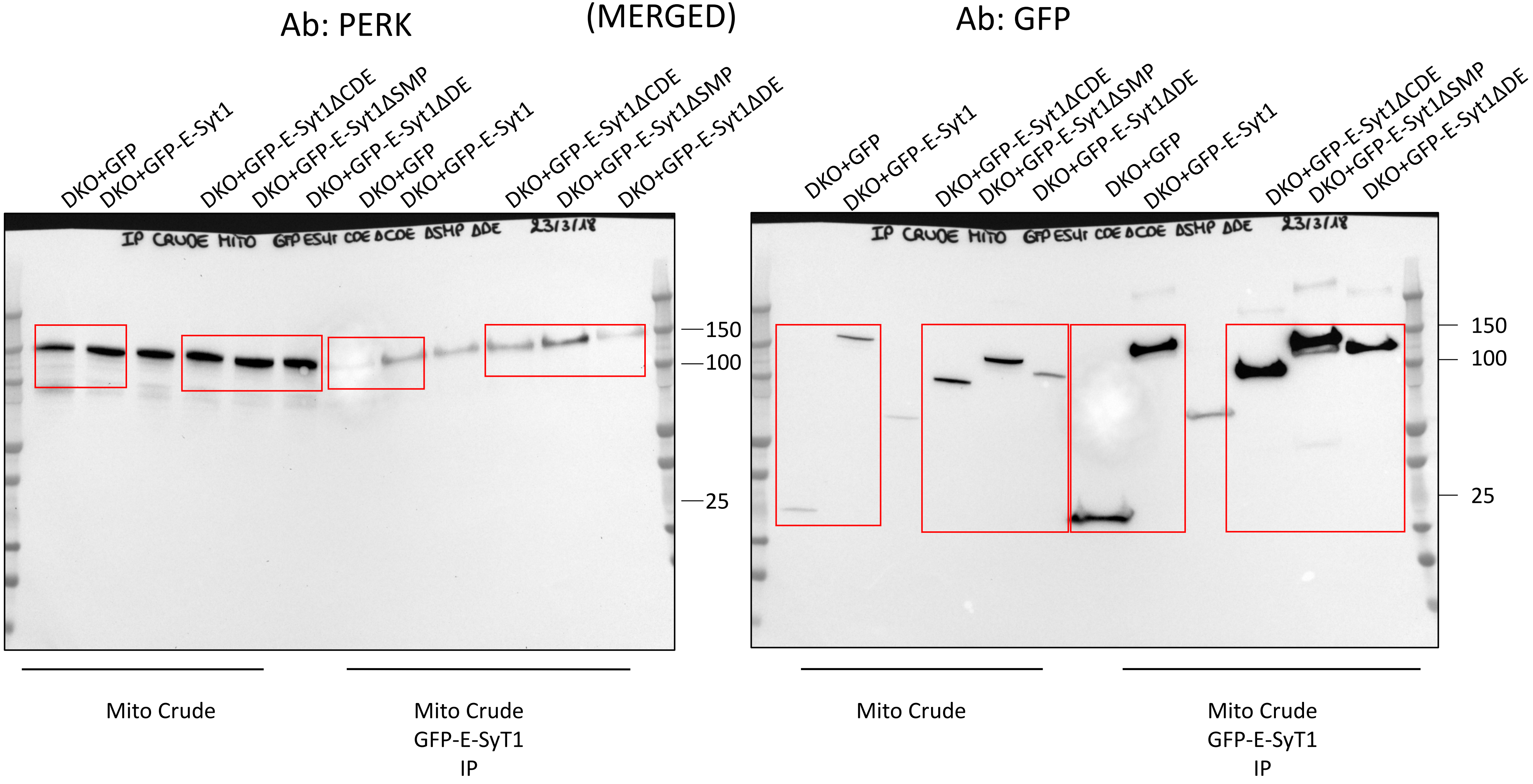

SourceData4
(PERK/GFP NOT merged with Ladder)

C

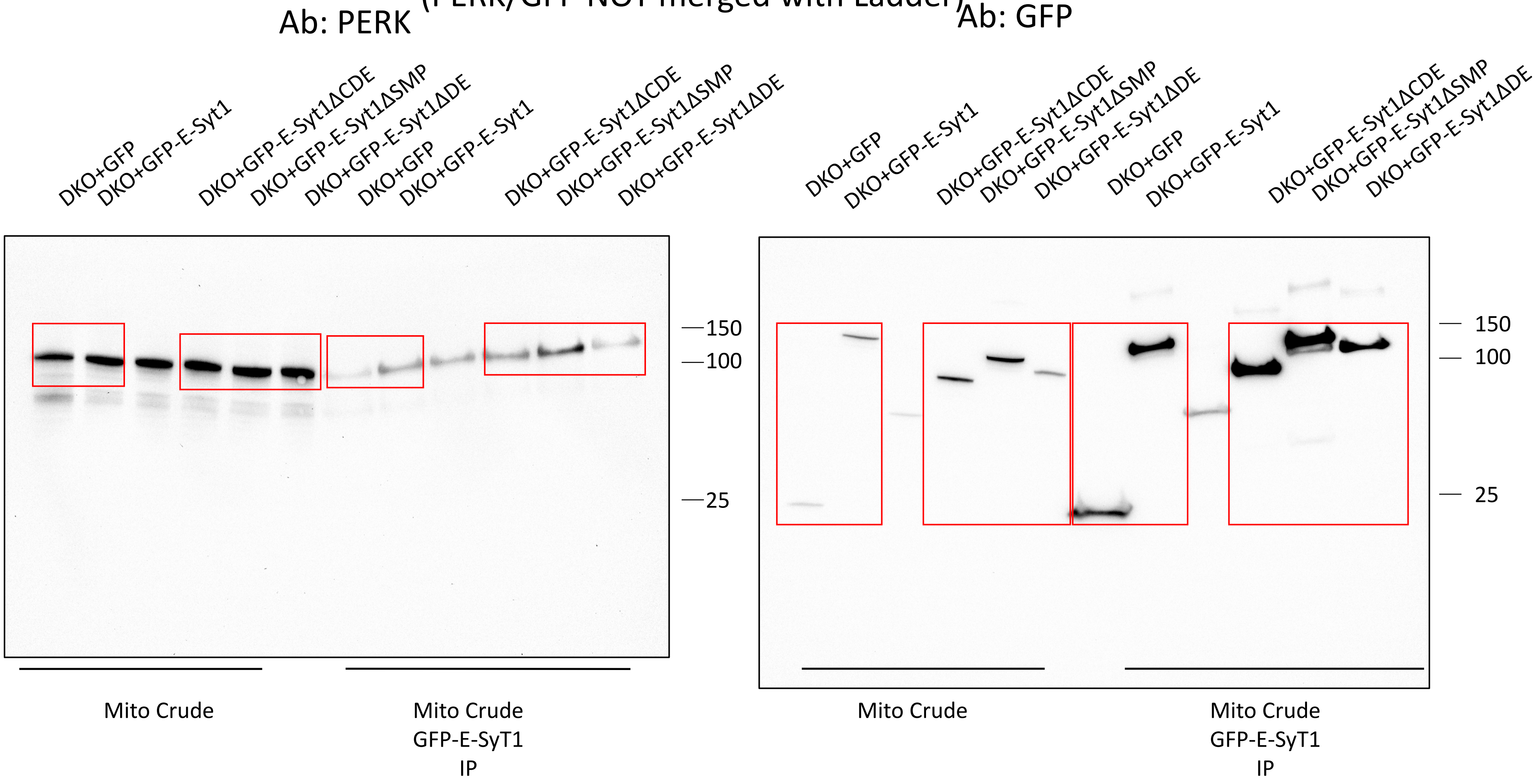

C

SourceData4
(only Ladder)

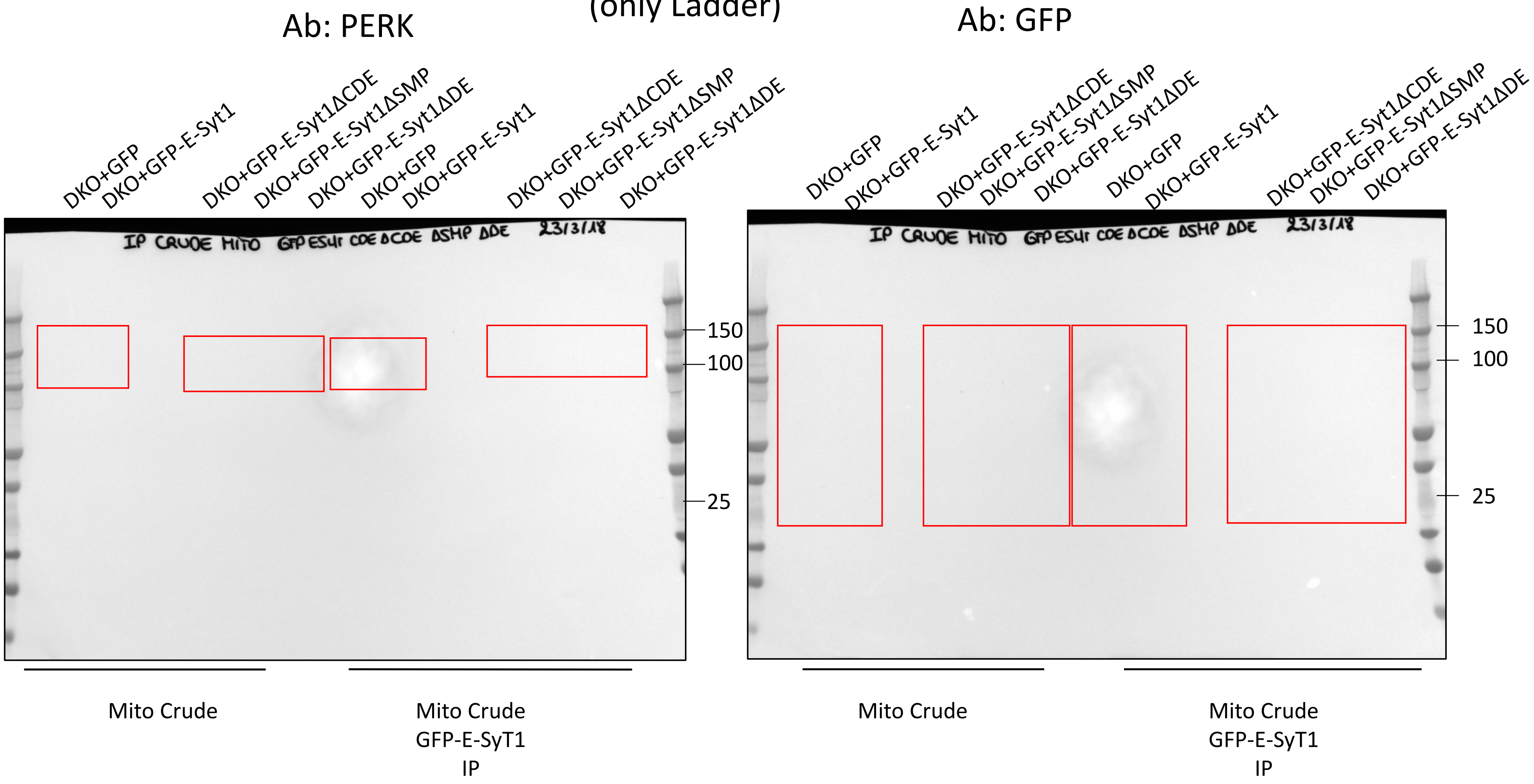

Supplement: SourceData F4 — is the source file for Fig. 4. [file JCB_202206008_SourceDataF4.pdf]
