# Supplementary material for: PERK recruits E-Syt1 at ER–mitochondria contacts for mitochondrial lipid transport and respiration
Source: J Cell Biol. 2023 Feb 23;222(3):e202206008. doi: 10.1083/jcb.202206008 (PMC9998969; doi:10.1083/jcb.202206008)

SourceData2S

A

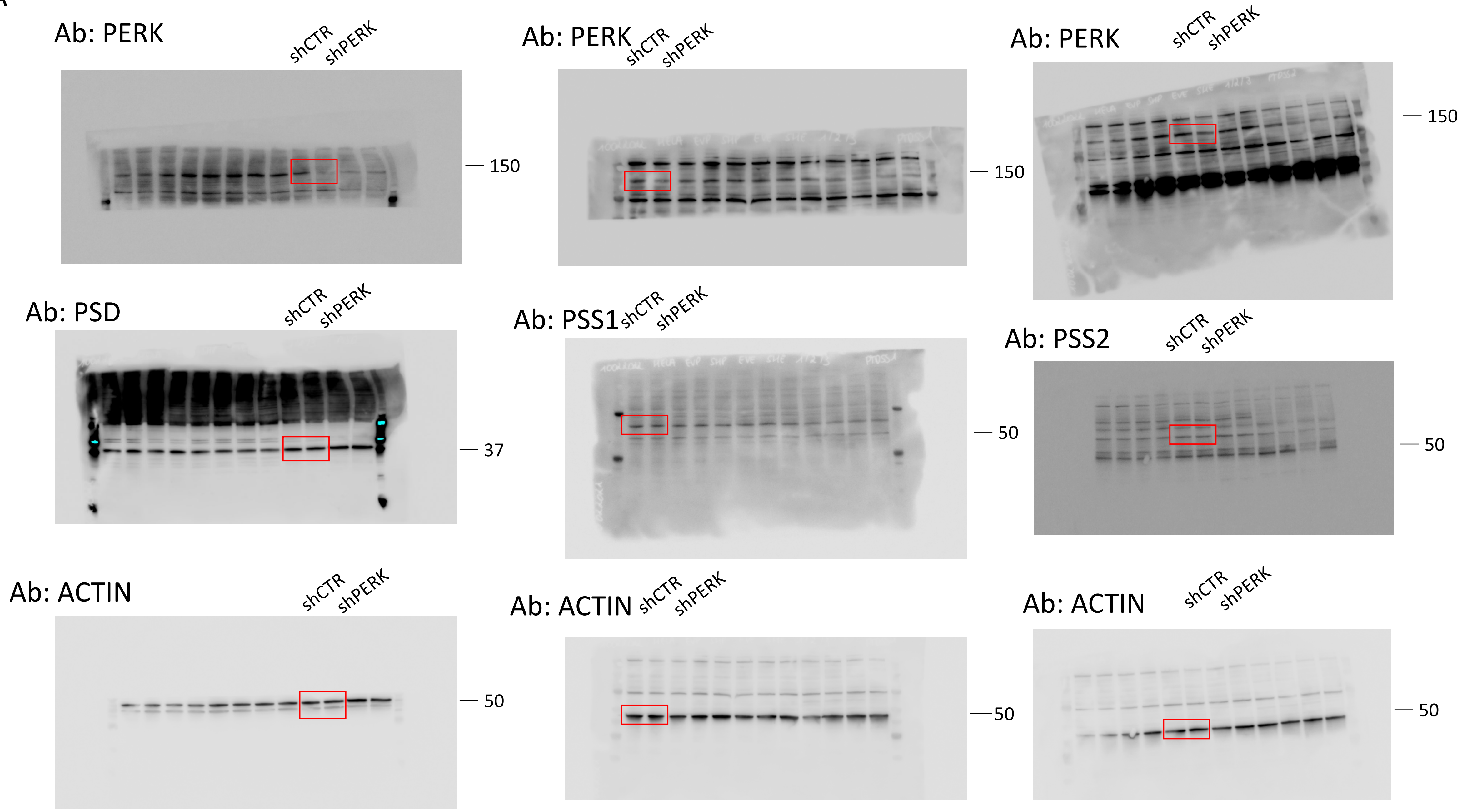

C

SourceData2S

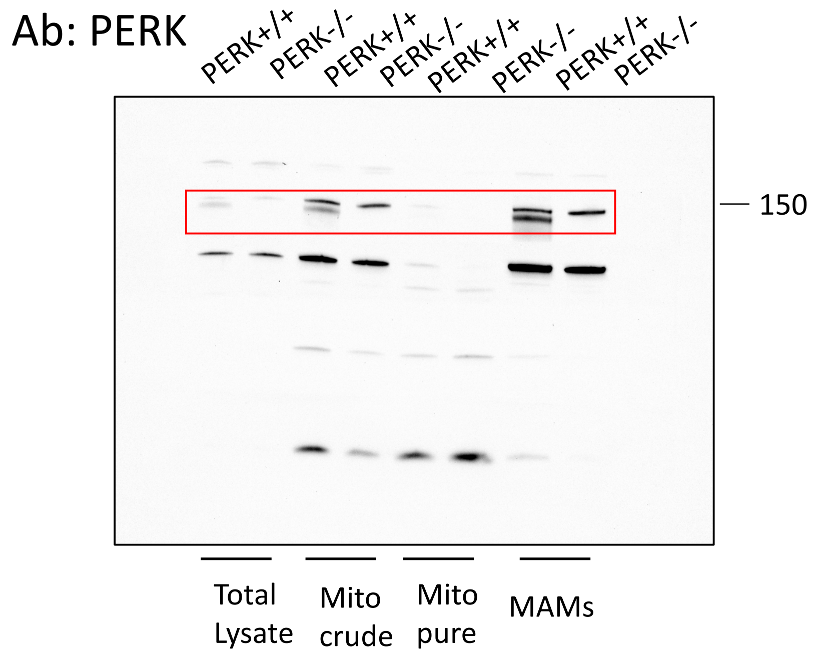

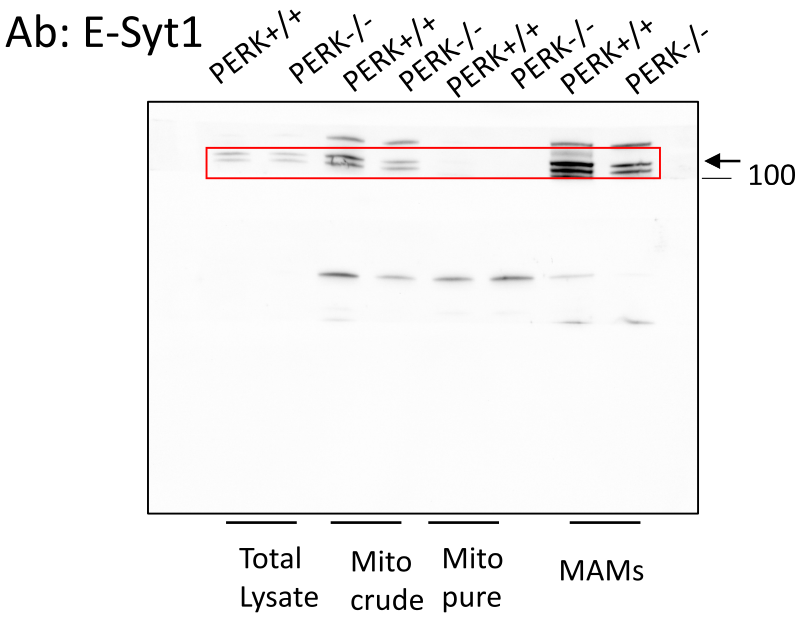

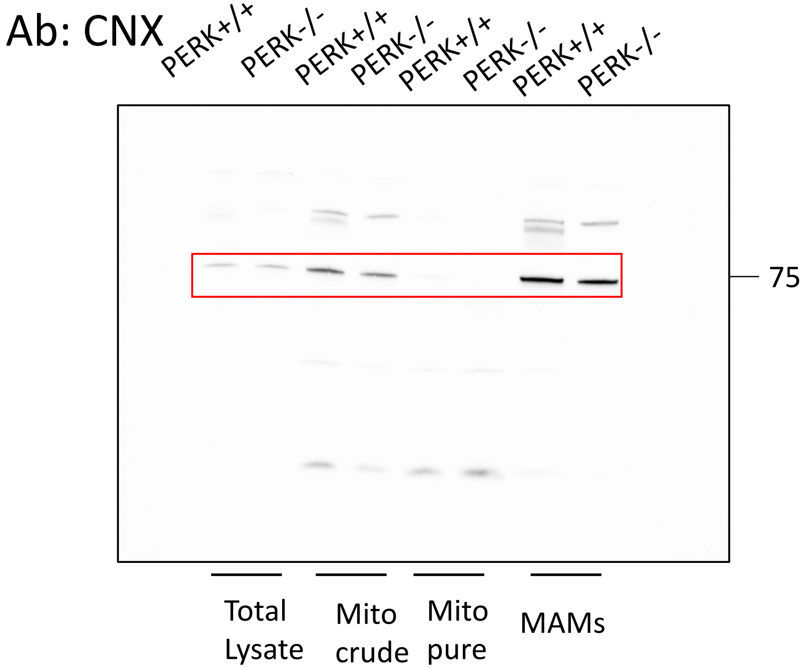

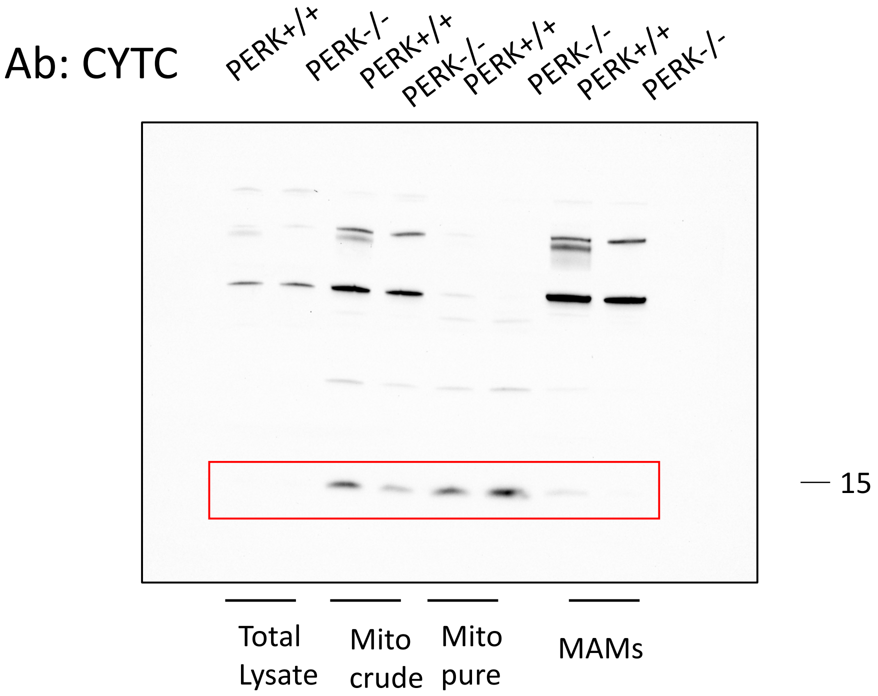

Supplement: SourceData FS2 — is the source file for Fig. S2. [file JCB_202206008_SourceDataFS2.pdf]
