# Supplementary material for: PERK recruits E-Syt1 at ER–mitochondria contacts for mitochondrial lipid transport and respiration
Source: J Cell Biol. 2023 Feb 23;222(3):e202206008. doi: 10.1083/jcb.202206008 (PMC9998969; doi:10.1083/jcb.202206008)

E

SourceData4S
(MERGED)

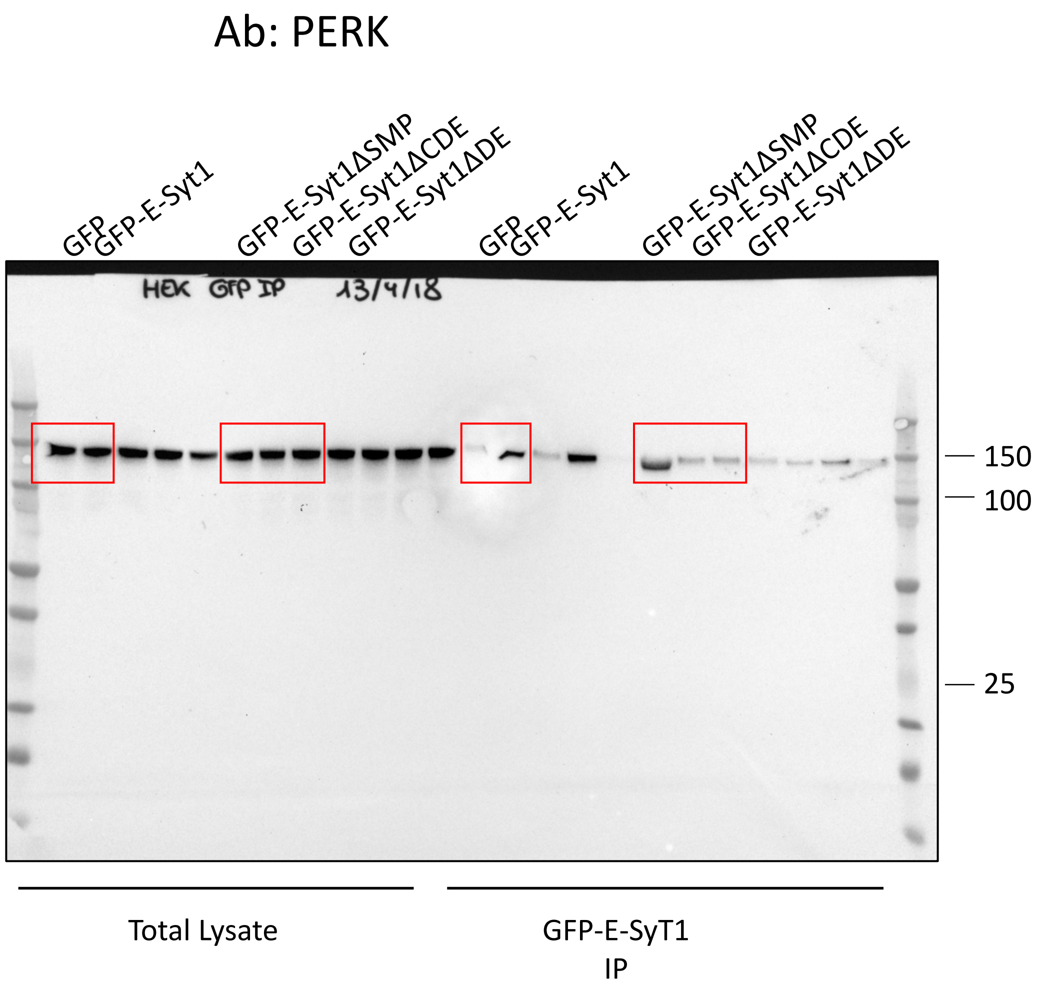

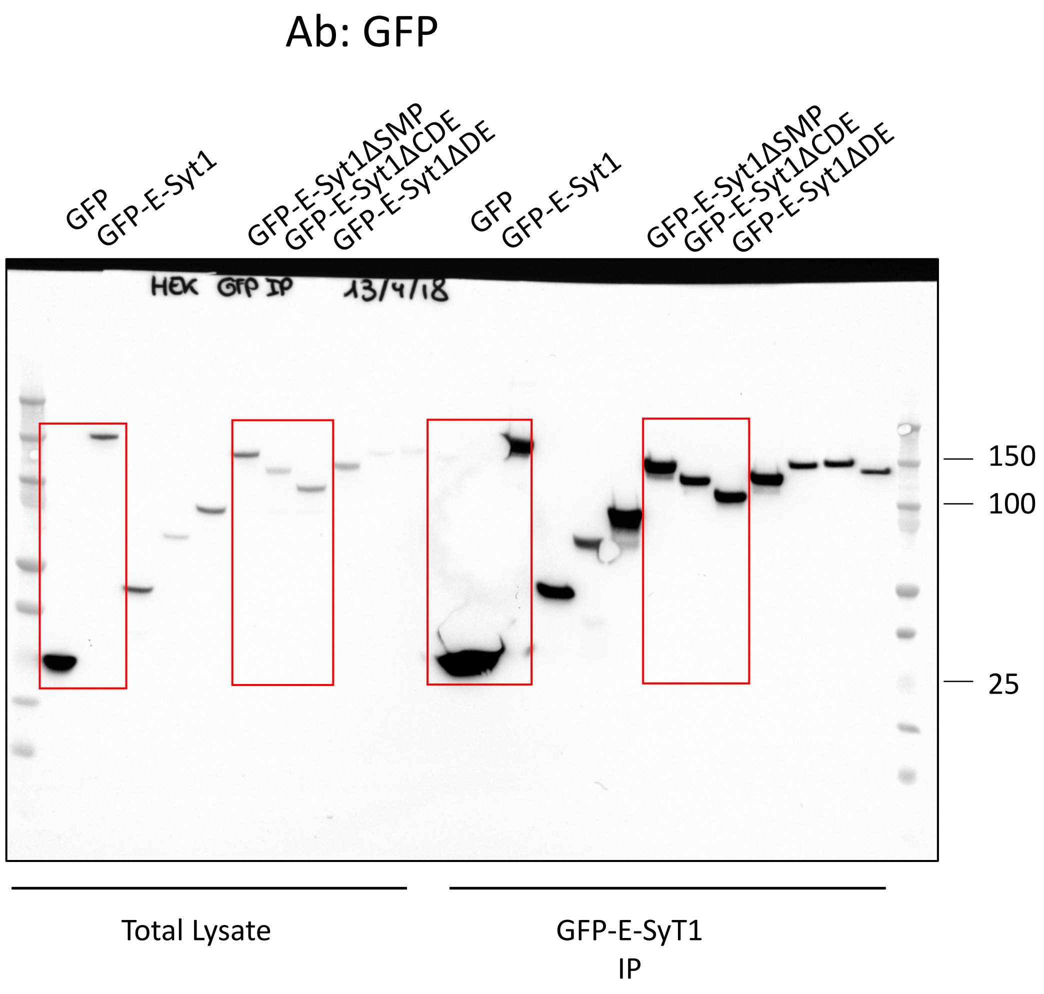

E

SourceData4S
(PERK/GFP NOT merged with Ladder)

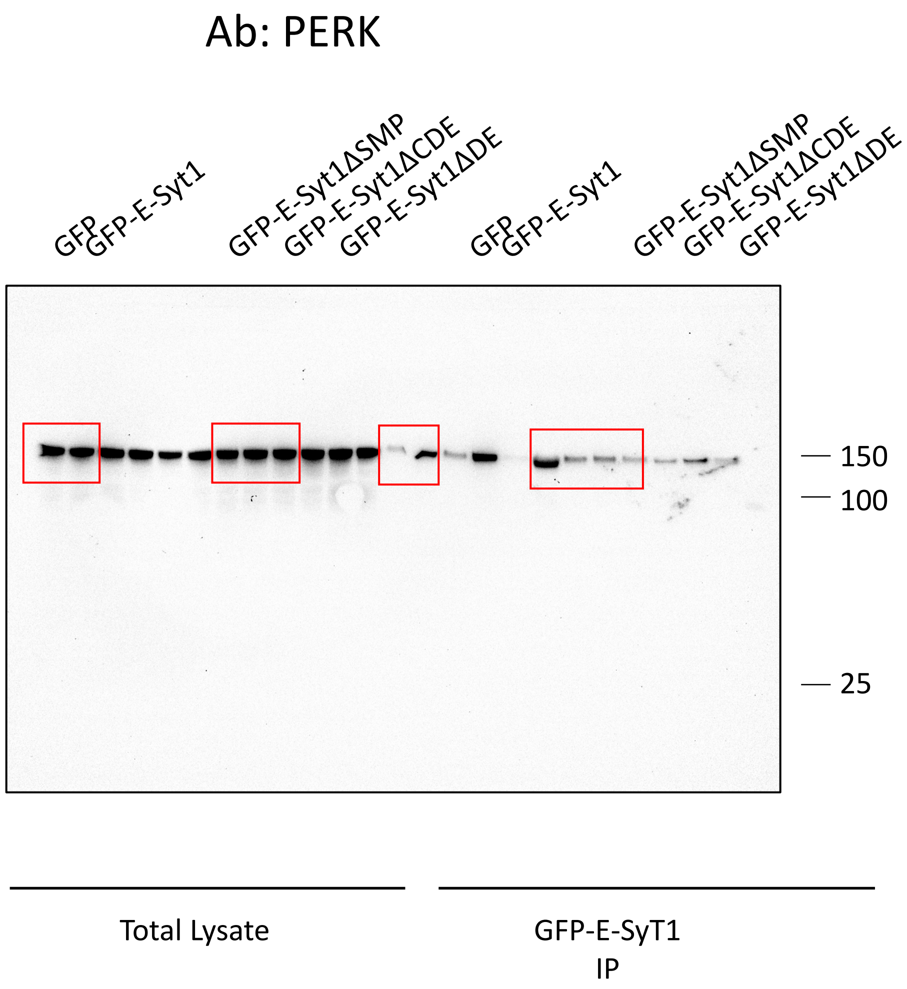

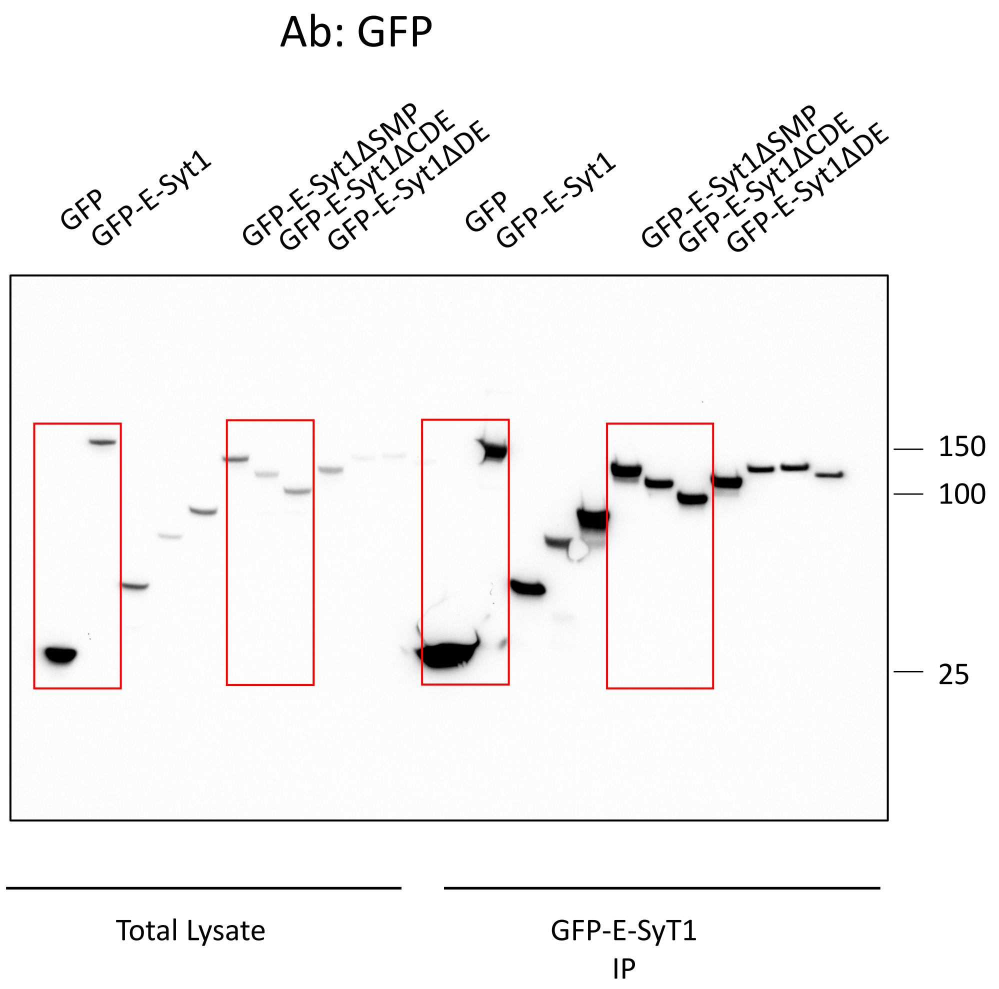

E

SourceDataS4
(only ladder)

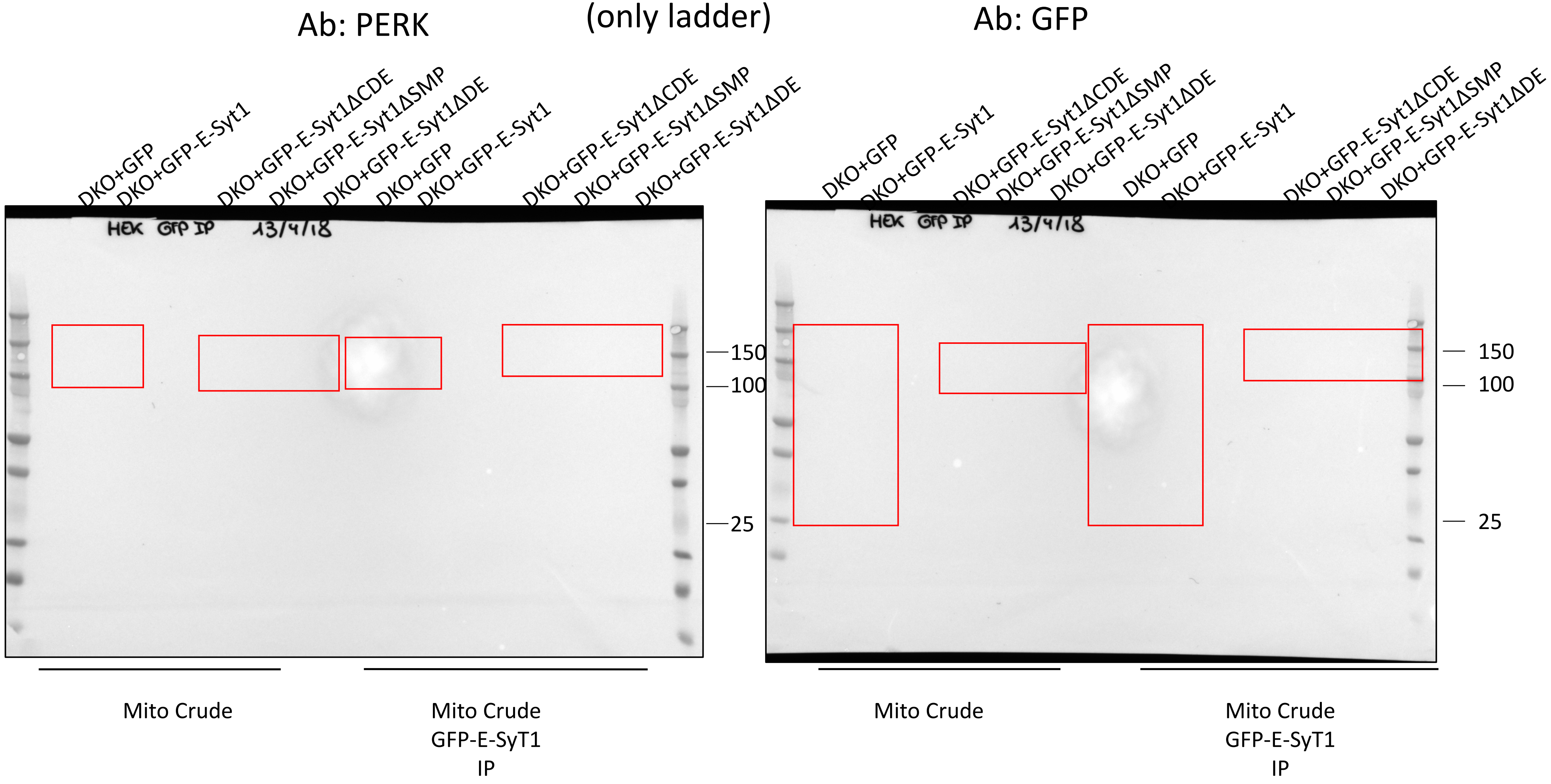

EXTRA EXAMPLE
(MERGED)

Ab: PERK(low Brightness&Contrast)

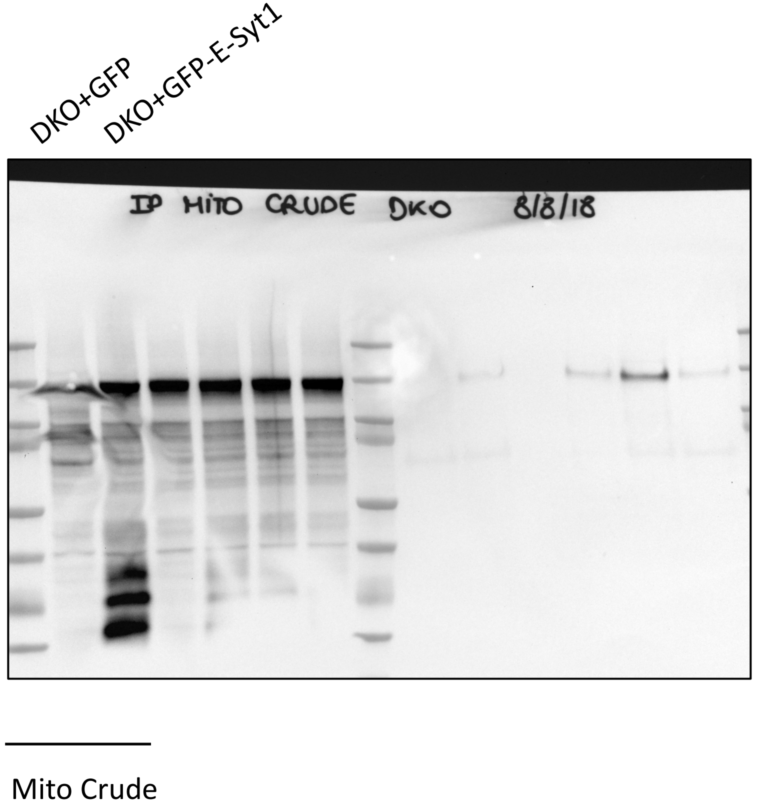

Ab: PERK(higher Brightness&Contrast)

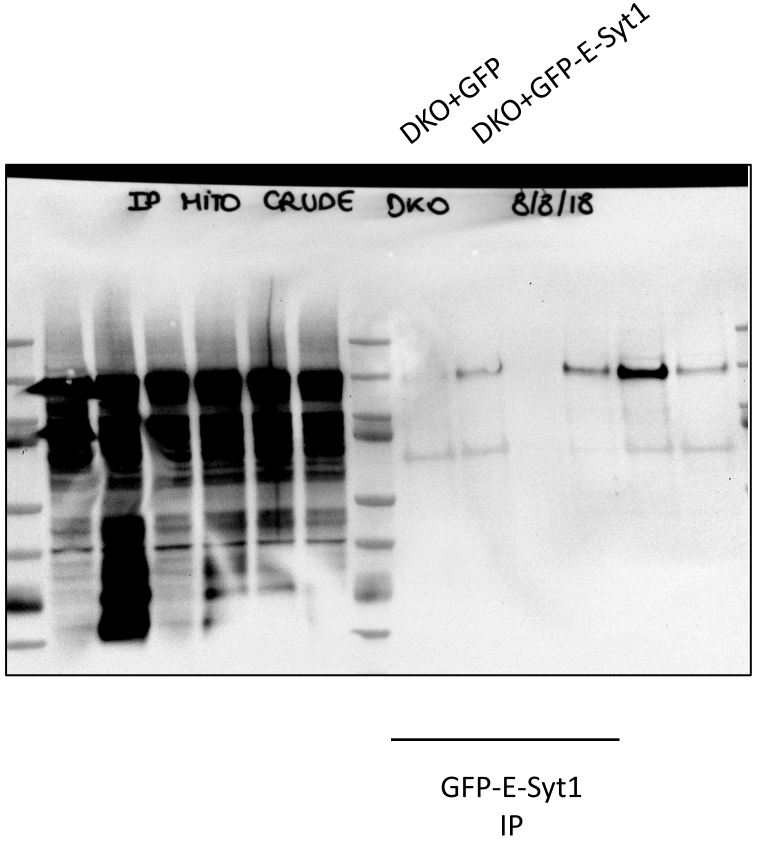

Ab: GFP

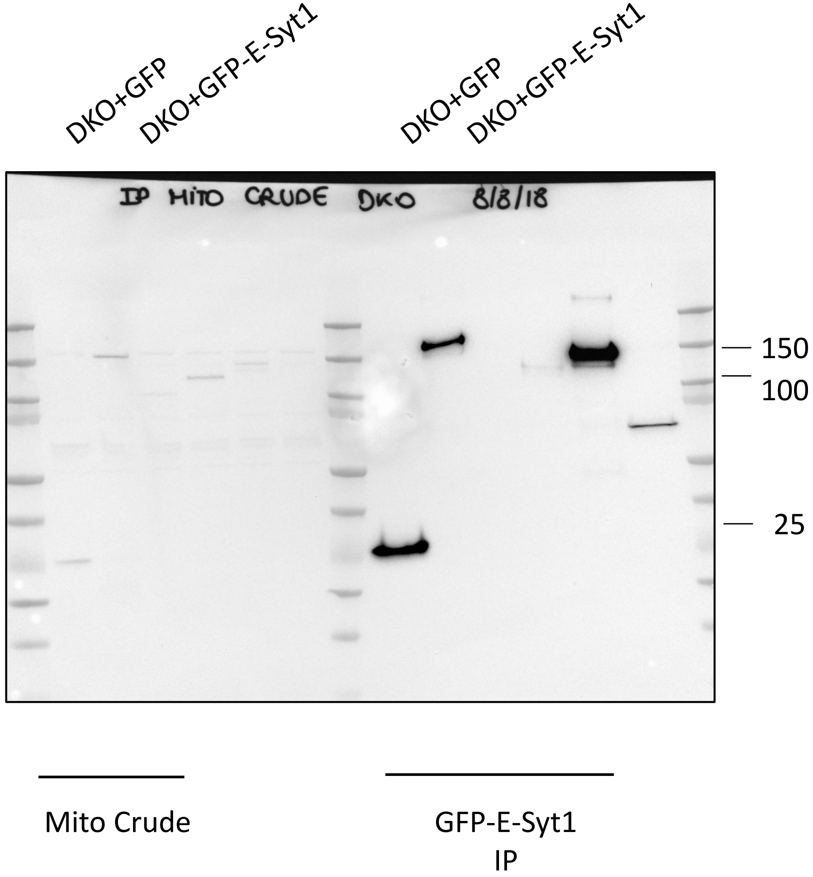

A

EXTRA EXAMPLE
(PERK/GFP NOT merged with Ladder)

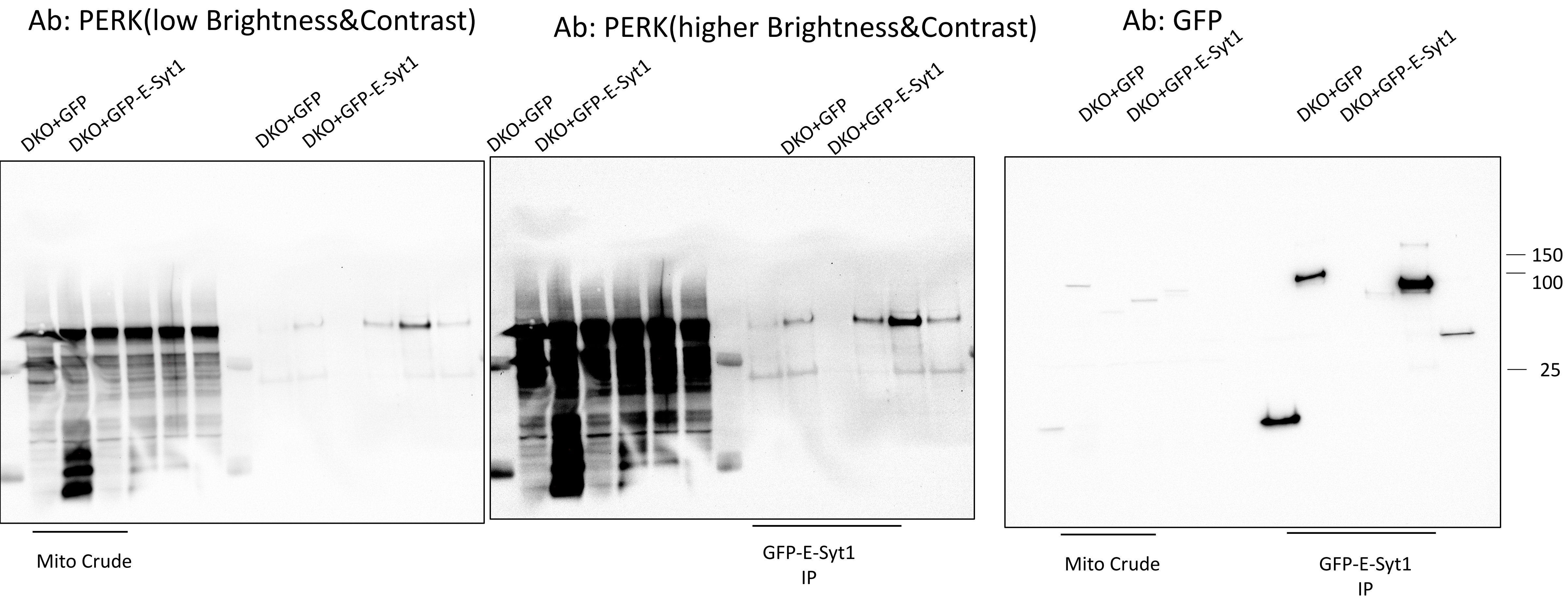

EXTRA EXAMPLE
(only Ladder)

A

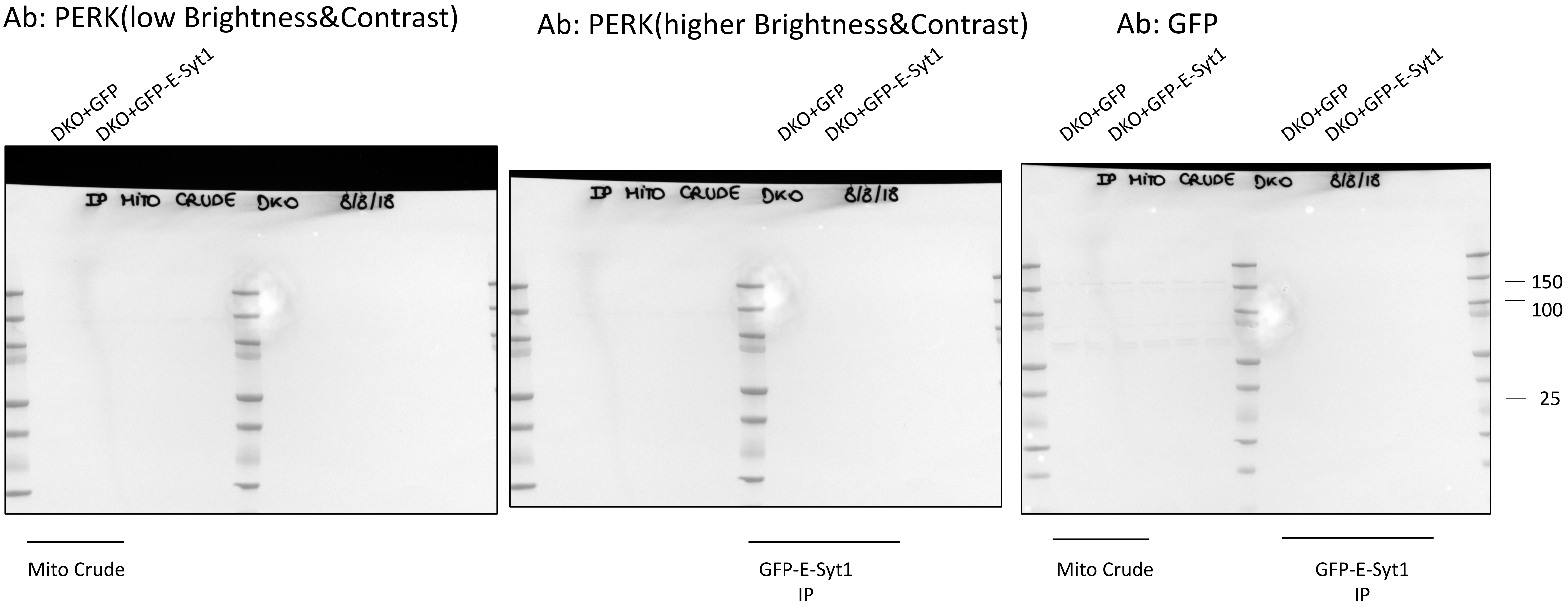

Supplement: SourceData FS4 — is the source file for Fig. S4. [file JCB_202206008_SourceDataFS4.pdf]
